# Nuevos retos para la planificación en salud: el Plan Nacional de Cáncer en Chile

**DOI:** 10.26633/RPSP.2020.6

**Published:** 2020-01-14

**Authors:** Pablo Villalobos Dintrans, Felipe Hasen, Catalina Izquierdo, Sylvia Santander

**Affiliations:** 1 Universidad de Santiago Santiago Chile Universidad de Santiago, Santiago, Chile.; 2 Ministerio de Salud Santiago Chile Ministerio de Salud, Santiago, Chile.

**Keywords:** Neoplasias, planificación en salud, sistemas de salud, Chile, Neoplasms, health planning, health systems, Chile, Neoplasias, planejamento em saúde, sistemas de saúde, Chile

## Abstract

Este artículo describe el proceso de elaboración del Plan Nacional de Cáncer de Chile. Este proceso incluyó una etapa inicial de diagnóstico en la que se convocó a diversos actores relevantes en el tema, con el fin de recoger distintas perspectivas y propuestas. Más tarde, la información recopilada fue sistematizada y estructurada por el Ministerio de Salud a través de un plan de acción, en el que se detallan sus iniciativas, objetivos e indicadores asociados. El Plan se definió en función de cinco líneas estratégicas a partir de las cuales se desprenden todas las acciones propuestas para los siguientes 10 años.

El objetivo del artículo es describir el proceso de elaboración del Plan, con el fin de extraer diversas lecciones que pueden ser útiles para la elaboración de otros planes de características similares en Chile y otros países de la Región.

Las principales lecciones aprendidas tienen relación con la necesidad de establecer un diagnóstico claro que permita hacer propuestas basadas en la evidencia, y la importancia de realizar este tipo de planificación a través de un proceso participativo y con una mirada interdisciplinaria, que potencie la solidez de las propuestas y facilite su validación y sostenibilidad.

## Abstract (en)

This article describes the process of developing Chile’s National Cancer Plan. This process included an initial diagnostic stage in which various stakeholders were called on to provide different perspectives and proposals. Later, the compiled information was systematized and structured by the Ministry of Health in an action plan with detailed initiatives, objectives, and associated indicators. The Plan was developed along five strategic lines that guide all the proposed actions for the next ten years.

The objective of the article is to describe the process by which the Plan was developed, in order to draw several lessons that may be useful in the preparation of other similar plans in Chile and in other countries of the Region.

The main lessons learned involve the need for clear diagnoses to make evidence-based proposals, and the importance of conducting this type of planning through a participatory process and with an interdisciplinary approach, resulting in sounder proposals and facilitating their validation and sustainability.

## RESUMO

Este artigo descreve o processo de elaboração do Plano Nacional do Câncer no Chile. O processo incluiu uma fase inicial de diagnóstico, na qual foram convocadas várias figuras relevantes nessa área a fim de reunir diferentes perspectivas e propostas. Posteriormente, as informações coletadas foram sistematizadas e estruturadas pelo Ministério da Saúde por meio de um plano de ação no qual foram detalhadas as suas iniciativas e objetivos e os indicadores relacionados. O Plano foi definido em função de cinco linhas estratégicas que servem como base para todas as ações propostas para os próximos 10 anos.

O objetivo deste artigo é descrever o processo de elaboração do Plano, com a finalidade de extrair diversas lições que possam ser úteis para a elaboração de outros planos com características semelhantes no Chile e em outros países da Região.

As principais lições aprendidas foram a necessidade de estabelecer um diagnóstico claro, que permita fazer propostas baseadas em evidências, e a importância de realizar esse tipo de planejamento por meio de um processo participativo e com uma perspectiva interdisciplinar, o que aumenta a solidez das propostas e facilita a sua validação e sustentabilidade.

Como en muchos otros países, el cáncer es hoy uno de los principales retos para la salud en Chile. En 2016, el cáncer era la segunda causa de muerte a nivel nacional, superado solo por enfermedades del sistema circulatorio. Sin embargo, se espera que esta jerarquía se revierta, y para el año 2023 se proyecta que el cáncer será la primera causa de muertes en el país (1). En la actualidad, además, el cáncer representa la principal causa de la carga de enfermedad, medida con años de vida ajustados por discapacidad (2).

En este escenario, el Ministerio de Salud de Chile (MINSAL) inició la elaboración de un Plan Nacional de Cáncer (PNC) con el fin de abordar de manera sistemática y organizada este problema de creciente relevancia. El PNC fue oficialmente lanzado el 4 de diciembre del 2018.

Hoy en día, la planificación sanitaria constituye una herramienta fundamental para la elaboración de políticas de salud, en un entorno de limitados recursos, incluidos diagnósticos y planes de acción específicos para responder a las múltiples necesidades de la población (3, 4). En este contexto, la elaboración del PNC constituye un importante ejemplo al respecto. Este artículo busca identificar lecciones que puedan ser usadas para iniciativas similares en el futuro tanto en Chile como en otros países, considerando los nuevos desafíos que el cambio demográfico y epidemiológico imponen a los sistemas de salud.

## LA ELABORACIÓN DEL PLAN NACIONAL DE CÁNCER: DEFINICIONES E HITOS INICIALES

El PNC se alinea con iniciativas llevadas a cabo recientemente en otros países que también han decidido abordar el problema del cáncer desde una perspectiva sistémica. En general, todos los planes se justifican desde una necesidad del sector salud en hacer frente a indicadores que han ido empeorando en el tiempo y la adopción de estrategias que permitan reducir los impactos de la enfermedad a nivel nacional. Si bien muchas de estas iniciativas comenzaron a ser implementadas en países desarrollados (5-11) –algunos ya en una segunda etapa de implementación–, el incremento del cáncer como una de las principales causas de muerte en diversos contextos ha motivado en época reciente el diseño de planes nacionales en países como Qatar y Zimbabwe (12, 13), destacando que el abordaje del cáncer es hoy un desafío a nivel mundial.

En abril del 2018, el MINSAL conformó el Grupo de Trabajo Asesor en Materias de Cáncer (GTAMC), un cuerpo consultivo científico-técnico multidisciplinario, deliberativo e independiente, compuesto por 77 integrantes (cuadro 1).

**CUADRO 1.** Actores e instituciones participantes en el Grupo Asesor en Materias de Cáncer para la elaboración del Plan Nacional de Cáncer de Chile

Tipo de actor	N° de participantes^a^	Organizaciones representadas
Sociedades científicas	16	Sociedad Chilena de Cancerología Sociedad Chilena de Anatomía Patológica Sociedad Chilena de Oncología Médica Sociedad Chilena de Radioterapia Oncológica Sociedad Chilena de Hematología Sociedad Chilena de Medicina Paliativa Red Chilena de Oncología Integrada Corporación Nacional Autónoma de Certificación de Especialidades Médicas Asociación Chilena de Educación en Enfermería Asociación Chilena de Agrupaciones Oncológicas
Universidades	11	Pontificia Universidad Católica de Chile Universidad de Chile Universidad Diego Portales Universidad del Desarrollo
Centros de salud	20	Instituto Nacional del Cáncer Fundación Arturo López Pérez Clínica Alemana Hospital Regional de Talca Hospital Sotero del Río Hospital Clínico Universidad Católica Centro Médico Biosenda Hospital Carlos Van Buren Clínica Oncológica IRAM Hospital Calvo Mackenna Clínica Las Condes Centro Programa Nacional de Cáncer Infantil
Organizaciones de la sociedad civil	18	Fundación Chile Sin Cáncer Fundación Foro Nacional del Cáncer Consultora Be-Trust Fundación Vivir Más Feliz Comunidad Organizaciones Solidarias Corporación 5 al Día Chile Fundación Nuestros Hijos Fundación Cáncer Vida Fundación Yo Mujer Fundación GIST Fundación Casa Sagrada Familia Fundación Mieloma Múltiple
Instituciones públicas	6	Ministerio de Desarrollo Social Ministerio del Deporte Ministerio de Educación Comisión Nacional de Ciencias y Tecnología

***Fuente*:** elaboración propia

a La suma de la columna “N° de participantes” excede de 77, ya que algunos participantes reportaron más de una filiación institucional.

**FIGURA 1.**
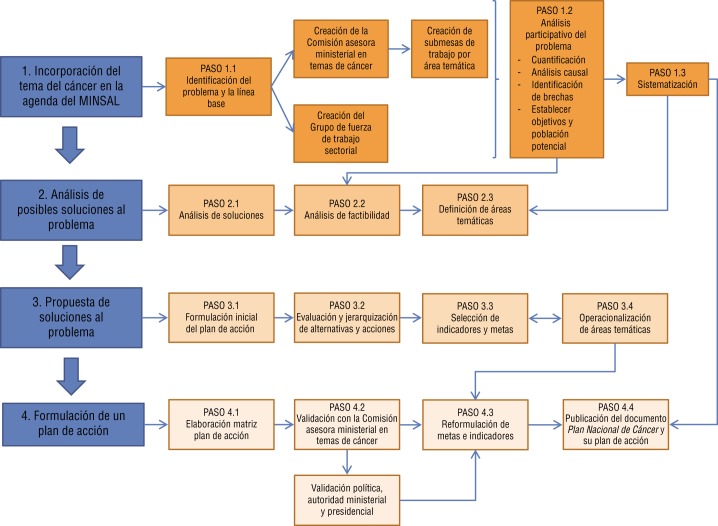
Flujograma del proceso metodológico para la elaboración del Plan Nacional de Cáncer de Chile

Esta instancia buscó ser un ente independiente que pudiera asesorar al MINSAL y recomendar acciones a realizar para avanzar hacia la disminución de la carga de esta enfermedad en el país (1). El establecimiento del GTAMC dio inicio al proceso de elaboración del plan (figura 1), dando comienzo a la etapa de “planificación sanitaria”, que considera un diagnóstico, identificación de soluciones y recursos, definición de objetivos y actividades, preparación de un presupuesto y diseño de un sistema que permita monitoreo y evaluación (3, 14).

## DEFINICIONES INICIALES: DIAGNÓSTICO Y PROPUESTAS

A través del trabajo del GTAMC y profesionales del MINSAL, se establecieron la visión, misión y áreas temáticas a ser trabajadas, y se estableció un período de diez años (2018-2028) para la implementación de las diversas estrategias del plan, siguiendo experiencias internacionales donde se proponen planificaciones de entre 5 a 10 años (5-13).

El trabajo del GTAMC se realizó con base en seis subcomisiones, con el objetivo de abordar con mayor profundidad las líneas estratégicas del plan y concentrar el trabajo de los expertos en temáticas de su interés y experticia, en un cronograma de trabajo desarrollado entre mayo y junio de 2018. Estas subcomisiones fueron:

- Capital humano.
- Prestaciones y provisión de servicios.
- Participación de la sociedad civil.
- Promoción, prevención y educación.
- Investigación e innovación.
- Registro.

El trabajo de las subcomisiones se desarrolló de manera independiente, con un coordinador central a cargo de convocar y dirigir las reuniones de trabajo y redactar los informes con diagnósticos y recomendaciones que cada mesa entregó al ministerio. Estos informes fueron luego sistematizados por el equipo técnico del MINSAL e incorporados como insumo para la elaboración del PNC y su respectivo Plan de Acción.

Además, se estableció una secretaría técnica en el MINSAL, encargada de recopilar la información y coordinar las reuniones de trabajo general del GTAMC ampliado.

De esta forma, dada la envergadura de las tareas necesarias para la elaboración del PNC, se conformó, al interior del MINSAL, un equipo multidisciplinario de profesionales, con la tarea de editar, sistematizar e incorporar las propuestas del GTAMC.

La primera etapa del trabajo se orientó a establecer un diagnóstico de la situación del cáncer en Chile, elemento clave para construir políticas públicas. Este diagnóstico consideró diversas dimensiones, como el número de afectados, las zonas geográficas, los costos sociales, los factores de riesgo y el análisis sociodemográfico.

En una segunda etapa, se trabajó en el análisis de soluciones propuestas por las subcomisiones de trabajo y se consideró la factibilidad presupuestal, socioeconómica, legal, política, ambiental y administrativa. En esta etapa, se establecieron los objetivos estratégicos para el PNC y su población potencial. Para esto, se documentó la información de las subcomisiones a través de pautas de trabajo, informes y acta de recomendaciones de los más de 70 expertos participantes, lo cual permitió acotar y encauzar las observaciones en el contexto de la opinión del conjunto.

MINSAL, Ministerio de Salud de Chile.

***Fuente*:** elaboración propia.

En la tercera etapa, se definieron las áreas temáticas relevantes para el PNC. Para cada línea temática propuesta por las comisiones, se elaboraron, junto con la asesoría metodológica de profesionales de MINSAL, –informes que permitieron definir las líneas estratégicas que permitieran operacionalizar estas recomendaciones.

**FIGURA 2.**
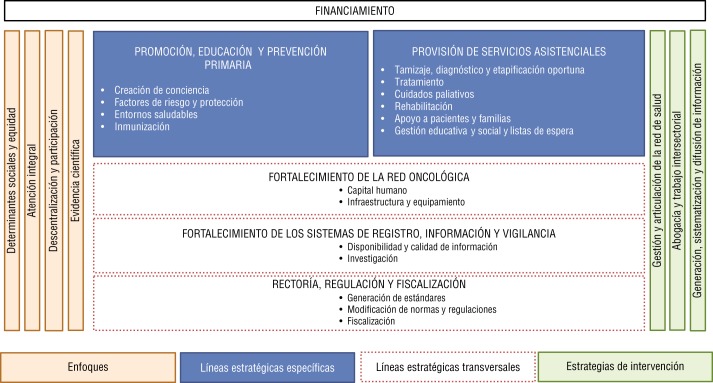
Matriz del plan de acción del Plan Nacional de Cáncer de Chile

Además de las líneas estratégicas, se propusieron varios enfoques integradores para el PNC, lo que permitió un abordaje integral e integrado con los distintos actores de la red de salud y otros actores sociales identificados (figura 2). Estos enfoques buscaban posicionar en la agenda pública al cáncer como un problema de salud prioritario y movilizar la acción del Estado, del individuo, la familia y la comunidad para el control de los factores de riesgo y las consecuencias individuales y sociales del cáncer.

Dichos enfoques se consideran transversales, complementarios e integradores, por lo que deben ser incorporados de manera permanente como herramienta de ayuda y, al mismo tiempo, como condición fundamental para una mejor calidad de la atención de salud y la efectividad de tratamientos, en concordancia con las recomendaciones de organismos internacionales y expertos nacionales.

### Operacionalización del Plan: elaboración de un marco y plan de acción

Toda la información generada en la etapa anterior se utilizó como insumo para operacionalizar las propuestas emanadas de las definiciones iniciales, proponiendo una estructura que contuviera estos aportes y que, a su vez, facilitara la implementación del Plan a los equipos del ministerio.

La propuesta se construyó con base en dos premisas: en primer lugar, que tuviera un horizonte en el corto, mediano y largo plazo y, en segundo lugar, que permitiera coordinar el trabajo al interior de las distintas unidades del MINSAL, así como con otros actores externos.

Respecto al horizonte de tiempo, se buscaba contar con un documento de referencia para comunicar los avances esperados en materia de cáncer en el sistema de salud y que, además, pudiera ser utilizado como carta de navegación para las políticas de salud en el corto, mediano y largo plazo. Mientras algunas acciones permitirían mejoras de corto plazo en la atención y provisión de servicios, otras, como el fortalecimiento de la red oncológica nacional, implicaban el desarrollo de proyectos en el mediano y largo plazo. Por esto, el PNC se diseñó con un horizonte de tiempo de diez años, similar a lo realizado por otros países (5-13) y, de esta manera, fijó una guía para las políticas nacionales en el tema, independiente del ciclo político de cada gobierno (cuatro años en Chile).

***Fuente*:** elaboración propia.

Por otra parte, se pensó en una estructura que explicitara la necesidad de un trabajo coordinado al interior del MINSAL, así como la participación de otros actores fuera del sector salud. Para esto, se elaboró un marco conceptual basado en líneas estratégicas, áreas y objetivos (figura 2), en línea con los modelos de planificación sanitaria (3, 4) y similar a las experiencias llevadas a cabo en otros países (9).

Dicho esquema propone cinco áreas estratégicas, dos consideradas específicas (“Promoción, educación y prevención primaria” y “Provisión de servicios asistenciales”) y que representan el *core* del Plan, mientras que el resto de las líneas (“Fortalecimiento de la red oncológica”, “Fortalecimiento de los sistemas de registro, información y vigilancia” y “Rectoría, regulación y fiscalización”) se definen como transversales y recogen objetivos que no representan un fin en sí, pero que son fundamentales para el cumplimiento de los objetivos de las líneas específicas. Además, estas líneas se relacionan de manera directa con la estructura del MINSAL, lo que le otorga al Plan un carácter transversal que incluye la labor de todas sus divisiones administrativas (15, 16).

A la izquierda de la figura 2 se muestran los enfoques del PNC; esto es, las directrices que guían el diseño y permean todas sus acciones. Estos enfoques no son objetivos que se intentan cumplir, sino que representan el espíritu que subyace a la propuesta del PNC y, por lo tanto, son transversales a todas las propuestas.

A la derecha, se detallan las estrategias de intervención; es decir, las formas en las que el ministerio pretende llevar a cabo las acciones propuestas en el PNC. Estas se dividen en tres grupos:

- Generación, sistematización y difusión de información (p. ej., creación de material, campañas, estudios).
- Consejo asesor en materias de cáncer, participación, abogacía y trabajo intersectorial (actividades que requieren coordinación con actores fuera del sector salud).
- Gestión y articulación de la red de salud (p. ej., generación o modificación de programas o iniciativas).

Por último, el esquema explicita la necesidad de contar con financiamiento para el desarrollo de las actividades del PNC como condición necesaria para su implementación.

Una vez establecido este marco, se realizó la sistematización de las propuestas emanadas de las comisiones. Para cada línea estratégica se identificaron áreas de trabajo (figura 2) con sus respectivos objetivos, las que a su vez incluyeron una o más iniciativas concretas que se propusieron para trabajar. Para estos, se definieron indicadores de proceso, resultado y actividades concretas a realizar, lo que permitió la elaboración de un gráfico de Gantt e indicadores para su monitoreo y evaluación.

Lo anterior generó un cambio de paradigma respecto de la manera en que tradicionalmente se ha trabajado a nivel ministerial en Chile. Se pasó de un esquema de trabajo en silos, definidos por áreas temáticas y en función de programas, a uno colaborativo en el cual distintas unidades ministeriales tributan a objetivos comunes. Esto permitió, por ejemplo, contar con un presupuesto asociado a la implementación del PNC, entendido como una iniciativa amplia, que aúna diversos objetivos, comprende diversos programas y coordina el quehacer de varios actores, tanto dentro como fuera del MINSAL.

Como etapa final, el documento incluyó un proceso de validación tanto interna (al interior del MINSAL) como externa (con expertos).

### Lecciones aprendidas

La formulación del PNC busca dar soluciones a largo plazo a un problema ya identificado y que requiere un enfoque intersectorial para su funcionamiento. Muchos de los aprendizajes sistematizados durante este proceso son aplicables no sólo para el abordaje del cáncer, sino también para sortear los obstáculos propios del quehacer de todas las políticas públicas en salud y otras áreas.

El desarrollo del PNC incorporó un trabajo para abordar los nudos críticos del proceso, con especial énfasis en el diagnóstico inicial y análisis de factibilidad de las soluciones, considerando su validación política, científica y ciudadana. A modo de resumen, se plantean tres importantes desafíos que enfrentaron los equipos técnicos y políticos, y las soluciones adoptadas.

### Estrategias basadas en evidencia

La realización del diagnóstico inicial se vio dificultada por los problemas de información, sobre todo la falta de registros poblacionales en cáncer, fundamentales para una toma de decisiones basada en la mejor evidencia disponible.

Las características de los registros ministeriales y la evaluación de la evidencia como un aporte al proceso de toma de decisiones en políticas requirieron de un importante trabajo de sistematización y validación de bases de datos relacionados al cáncer en Chile. Así, se redoblaron los esfuerzos para garantizar que este proceso y la información resultante fuesen correctamente identificados, evaluados y utilizados por todos los actores que intervinieron en la elaboración del Plan.

Fortalecer el uso de la evidencia en la construcción de políticas públicas y avanzar hacia la sistematización de datos a nivel nacional son desafíos clave para la implementación de cualquier iniciativa en esta línea.

### Metodología participativa

Si bien la incorporación de metodologías de participación y co-construcción efectiva de políticas públicas junto a distintos actores sociales implica un mayor esfuerzo técnico, metodológico y político, este enfoque también permite una mayor validación y legitimidad del resultado final (17, 18). Al respecto, la construcción del PNC realizó un trabajo colaborativo donde diversos actores sociales, institucionales y políticos dieron su aporte.

En lugar de imponer los estándares y valores ministeriales e institucionales al PNC, el proceso de participación y co-construcción buscó generar un espacio que permitiera conocer, reconocer y respetar las necesidades y propuestas de distintas partes interesadas en el tema del cáncer, y así generar mecanismos para el intercambio de información, reflexión, análisis y elaboración de soluciones desde lo local y territorial, lo intersectorial y la interdisciplina, la academia y la política, para formar y fortalecer redes y alianzas estratégicas.

En este contexto, la estrategia de participación colaborativa permitió una labor conjunta entre los profesionales de salud, los equipos técnicos, académicos, organizaciones de la sociedad civil y autoridades políticas, con el establecimiento de prioridades, mecanismos de acción y evaluación, y la reflexión de todos los participantes en torno al cáncer como un problema de salud pública.

### Trabajo interdisciplinario

Otro desafío importante en el proceso del PNC fue la incorporación de un equipo multidisciplinario en su elaboración. Así, se entiende que los problemas cada vez más complejos que enfrentamos hoy, en especial en salud pública, requieren del aporte de profesionales de distintas áreas para avanzar en su solución. En esta línea, el Plan se vio enriquecido por el aporte de profesionales de áreas como salud, antropología, economía, psicología, administración y gestión, entre otros.

De esta forma, el proceso de elaboración del PNC permitió la integración de distintos conocimientos y prácticas desde diversas disciplinas, y generar de esta manera una propuesta técnicamente sólida, para incrementar su implementación exitosa y sostenibilidad futura.

A partir de la información presentada, el artículo permite extraer importantes lecciones para Chile y otros países que se embarquen en similares procesos de reforma y planificación en salud. Se destaca la utilidad de construir una estrategia amplia que permita una mirada holística a un problema de salud particular, incluida la participación de diversos actores dentro y fuera del sector salud. Lo anterior permite un trabajo más coordinado, fomentar la cooperación intersectorial e incrementar la posibilidad de éxito y sostenibilidad de las propuestas.

### Contribuciones de los autores.

PVD, FH, CI y SS concibieron el estudio original y escribieron el manuscrito original. Todos los autores revisaron y aprobaron la versión final.

### Agradecimientos.

Los autores reconocen la participación de diversos actores en el proceso de elaboración del Plan; sin su aporte, ni el Plan Nacional ni este artículo hubieran sido posible. Se agradecen los comentarios de tres revisores anónimos y un editor de la revista, cuyos aportes contribuyeron a mejorar la versión inicial del documento.

### Declaración.

Las opiniones expresadas en este manuscrito son únicamente responsabilidad de los autores y no reflejan necesariamente los criterios ni la política de la *RPSPP/AJPH* o la Organización Panamericana de la Salud.
